# Molecularly Imprinted Polymers and Surface Imprinted Polymers Based Electrochemical Biosensor for Infectious Diseases

**DOI:** 10.3390/s20040996

**Published:** 2020-02-13

**Authors:** Feiyun Cui, Zhiru Zhou, H. Susan Zhou

**Affiliations:** Department of Chemical Engineering, Worcester Polytechnic Institute, 100 Institute Road, Worcester, MA 01609, USA; fcui@wpi.edu (F.C.); zzhou4@wpi.edu (Z.Z.)

**Keywords:** molecularly imprinted polymers (MIPs), surface imprinted polymers (SIPs), electrochemical biosensor, biomarkers for infectious diseases

## Abstract

Owing to their merits of simple, fast, sensitive, and low cost, electrochemical biosensors have been widely used for the diagnosis of infectious diseases. As a critical element, the receptor determines the selectivity, stability, and accuracy of the electrochemical biosensors. Molecularly imprinted polymers (MIPs) and surface imprinted polymers (SIPs) have great potential to be robust artificial receptors. Therefore, extensive studies have been reported to develop MIPs/SIPs for the detection of infectious diseases with high selectivity and reliability. In this review, we discuss mechanisms of recognition events between imprinted polymers with different biomarkers, such as signaling molecules, microbial toxins, viruses, and bacterial and fungal cells. Then, various preparation methods of MIPs/SIPs for electrochemical biosensors are summarized. Especially, the methods of electropolymerization and micro-contact imprinting are emphasized. Furthermore, applications of MIPs/SIPs based electrochemical biosensors for infectious disease detection are highlighted. At last, challenges and perspectives are discussed.

## 1. Introduction

Infectious diseases can be disseminated widely in various ways. They are mainly caused by pathogenic microorganisms, such as viruses, bacteria, fungi, or parasites. Despite great achievements in diagnosis, treatment, and prevention, infectious diseases remain a serious global health threat [1,2]. The challenges of controlling infectious diseases include irrational use of antibiotics, an increase of multidrug-resistant pathogens, the emergence of new pathogenic microorganisms, and rapid spread owing to globalization and overpopulation [3]. Timely diagnosis and targeted antimicrobial treatment are important for the successful clinical control of infectious diseases. Current diagnostic methods for infectious diseases mainly rely on laboratory-based tests including culture, microscopy, enzyme-linked immunosorbent assay (ELISA), and polymerase chain reaction (PCR) [4]. These methods are time-consuming, expensive, and required to be operated by a specialist. Biosensors are ideal alternative methods for timely diagnosis of infectious diseases. They have many merits such as high sensitivity, quick read-out time, and are easier to be mass fabricated and miniaturized. They also can be used as point-of-care (POC) devices at a doctor’s office or home because of their simplicity and affordability. Therefore, extensive research has been published to report ultrasensitive electrochemical biosensors for infectious disease detection with excellent performance.

**Figure 1:**
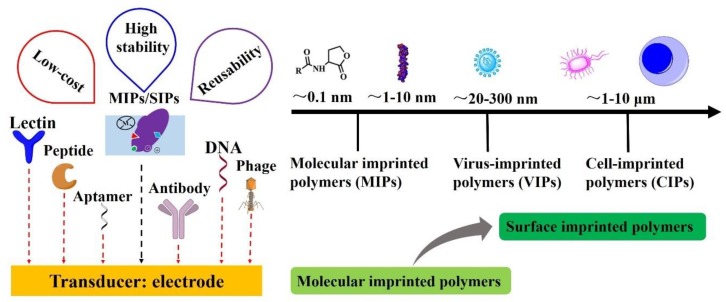
Various receptors for electrochemical biosensors applied in infectious diseases biomarker detection and size distribution covering all the analytes in this review, including small molecular, toxin protein, virus, bacteria, and fungal cells as plotted on a nanometer scale chart.

Receptors and transducer are the two main components of biosensors. The receptor recognizes the analyte specifically and the transducer converts the binding activity into a measurable signal sensitively. Electrodes are used as a transducer in electrochemical biosensors [5]. Natural receptors (Figure 1) such as antibodies, DNA, aptamer, phage, lectin, and peptide are used as receptors. They have a high affinity to their targets, but there are also huge challenges in practical applications because of their poor durability and repeatability at high temperature, pressure, in organic solvents, and also low stability in low or high pH solutions. Alternatively, the molecular imprinting technique has been reported to overcome most of these drawbacks. Molecularly imprinted polymers (MIPs) [6] and surface imprinted polymers (SIPs) [7,8] have a great potential to be robust artificial receptors (also called plastic antibodies) [9]. Due to its chemical and physical stability, MIPs/SIPs have provided a new insight for creating receptors by forming specific cavities for binding analytes in the polymeric matrix. In contrast to natural receptors, MIPs/SIPs offer an inexpensive, rapid, sensitive, easy-to-use, and highly selective receptors for sensors, typically for the electrochemical biosensors. Hence, MIPs/SIPs based electrochemical biosensors have become very attractive for infectious diseases. 

Several related reviews have been reported. Lahcen et al. [10] mainly presented the development of MIPs modified with nanomaterials for electrochemical biosensors. Good electrical catalytic properties and excellent conductivity of nanomaterials combined with the comparable selectivity of MIPs endow them with powerful performance for various kinds of biomarkers. The magnetic nanoparticles, carbon dots, multi/single-walled carbon nanotubes, and graphene oxides modified MIPs for electrochemical sensing were highlighted in their review paper. Origins, preparation methods, and applications of SIPs applied in larger biomarkers were reviewed by Eersels and coworkers [11]. They pointed out that the measurement of larger biomarkers such as viruses, bacteria, or cells met challenges when using the classical MIPs concept. SIPs can form binding cavities directly on the surface of cured polymers, thus making it easier to remove the templates and provide better use in larger biomarkers (Figure 1). 

In this review, current trends in the development of MIPs/SIPs based electrochemical biosensors for rapid assessment of the infectious diseases, as well as future research directions are comprehensively summarized and discussed. Virus-imprinted polymers (VIPs) [12] for virus detection and cell-imprinted polymers (CIPs) [13] for bacteria detection are highlighted (Figure 1).

## 2. Recognition Mechanisms Between Imprinted Polymers with Biomarkers

**Figure 2:**
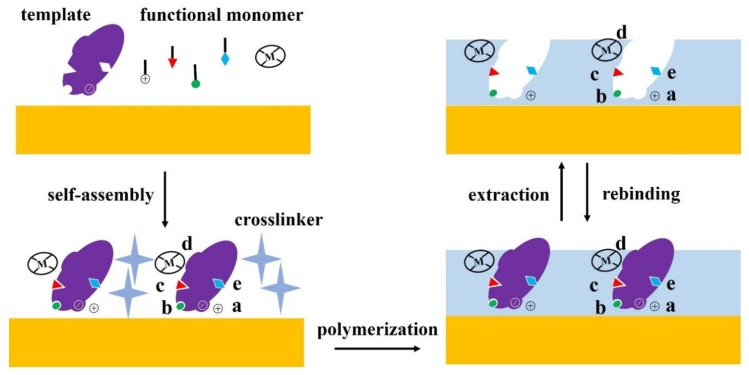
Preparation procedures of molecularly imprinted polymers (MIPs) and surface imprinted polymers (SIPs) on an electrode and various interactions of template (analyte) and MIPs/SIPs, (**a**) electrostatic interactions, (**b**) reversible covalent bonds, (**c**) van der Waals or hydrophobic interactions, (**d**) metal chelation, and (**e**) hydrogen bonds.

The size and morphology of cavities are critical factors for specific recognition between MIPs/SIPs and biomarkers. Besides these, chemical recognition of the biomarkers is important. Three types of chemical recognition methods have been reported: non-covalent, semi-covalent, and covalent. Because of its excellent adaptability, the non-covalent recognition that includes hydrogen bonds, hydrophobic, and electrostatic interactions is the most widely applied for the fabrication of MIPs/SIPs [14,15]. Figure 2 presents various interactions of the template (analyte) and MIPs/SIPs. 

### 2.1. Small Molecular Biomarkers

**Figure 3:**
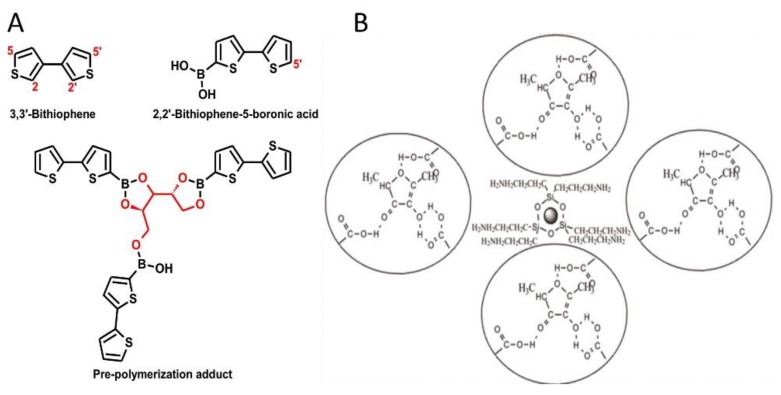
(**A**) Structural formulas of the d-arabitol template, 2,2′-bithiophene-5-boronic acid functional monomer, 3,3′-bithiophene crosslinker, and d-arabitol esterificated with three molecules of functional monomer. Reproduced from [18]—Published by the American Chemical Society. (**B**) Structural formulas of methacrylic acid (MAA) and 2,5-dimethyl-4-hydroxy-3(2H)-furanone (DMHF). Reproduced from [21]—Published by Elsevier B.V.

Metabolites and small signaling molecules produced by microorganisms can be used as biomarkers of infectious diseases. For example, both l- and d-arabitol can be produced by human cells as natural metabolites. They are normally almost equal amounts in healthy humans’ body fluids. However, only d-arabitol can be produced by fungi of the *Candida* family. Hence, excess of d-arabitol in body fluids can be used as a biomarker for the diagnosis of candidiasis [16,17]. Dabrowski et al. [18] developed electrochemical sensors based on MIPs for d-arabitol detection in urine samples of patients with candidiasis. They used 2,2′-bithiophene-5-boronic acid as a functional monomer because weak ester bonds can be formed by its boronic acid group and vicinal hydroxyl moieties of d -arabitol. The bithiophene group of 2,2′-bithiophene-5-boronic acid can be polymerized in position 5 of the thiophene ring (Figure 3A). The crosslinker 3,3′-bithiophene could be polymerized in its 2, 2′, 5, and 5′ four positions. The oxidation peak of the crosslinker and functional monomer was at ~1.45 V and ~1.10 V respectively with silver as a pseudo reference electrode. Hence, 0.50~1.20 V was used to induce the initiated polymerization to create a cation radical. Then the crosslinker 3,3′-bithiophene passively participated in the electropolymerization as an acceptor of the cation radical attack (Figure 3A). 

N-acyl-homoserine-lactones (AHLs) are important signal molecules of gram-negative bacteria. They participate in the quorum sensing (QS) system to induce and regulate the expression of virulence [19,20]. Jiang et al. [21] used methacrylic acid (MAA) as a monomer and 2,5-dimethyl-4-hydroxy-3(2H)-furanone (DMHF) as an analog template to construct the magnetic molecularly imprinted polymers (MMIPs) which have the capability to selectively recognize AHLs. The hydrogen bond and the delicate binding microcavities are the main contributors to the specificity (Figure 3B). 

### 2.2. Toxins and other Protein Biomarkers

Microbial toxins produced by microorganisms, including bacteria and fungi, are of high molecular weight and have antigenic properties. They can promote infectious diseases by directly damaging host tissues and disabling the immune system. Hence, the fast detection of microbial toxins is critical for the diagnosis of infectious diseases. Most of the microbial toxins are protein. For protein biomarkers, a simple way to improve the affinity of the target protein for its rebinding position is to locate specific charges at its specific rebinding site. A positively charged monomer, quaternary ammonium salt, holding a vinyl bond and an aromatic ring (VBTC), was used to assemble the MIPs for bovine serum albumin (BSA) which holds a negative charge under analytical conditions (pH 7.4, isoelectric point is 5.4). It promoted the ionic interaction between BSA and the MIPs [22]. π–π interaction was used to recognize toxic protein aflatoxin B1 by the p-aminothiophenol-based MIPs. The sensitivity of the imprinted sensor was 11 times greater than that of the non-imprinted sensor by applying the π-donor/π-acceptor interaction [23]. 

### 2.3. Virus

**Figure 4:**
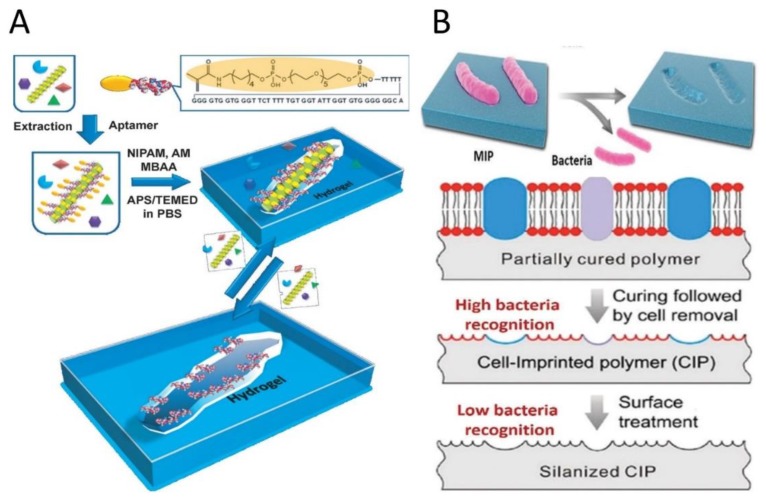
(**A**) Preparation process for virus sensitive super-aptamer hydrogels MIPs. Reproduced from [25]—Published by John Wiley and Sons. (**B**) Schematic diagram of cell-imprinted polymers for bacteria cells. Reproduced from [30]—Published by The Royal Society of Chemistry and [27]—Published by the American Chemical Society.

Creating virus-affinity MIPs/SIPs by imprinting techniques has great potential for the diagnosis of virus-related diseases. The most direct and simple method to prepare virus recognition sites is surface virus-imprinting. The viruses can be identified by their morphology and surface properties easily. Some molecules of the virus capsid are demonstrated to play a vital role in the chemical recognition between MIPs/SIPs and viruses [24]. Bai and Spivak developed a hydrogel-based SIP to recognize the Apple Stem Pitting Virus (ASPV). For preparing virus SIPs in the molecular-scale, the perhydro gel solution was fabricated by incubation of the impure ASPV extract with polymerizable ASPV-specific aptamers. Their results proved the need for aptamer preorganization by using the ASPV template, which illustrated the significance of the recognition mechanism for imprinting ASPV-specific sites (Figure 4A). Multivalent interactions of ASPV and aptamers-hydrogel based SIPs induce to evident visible volume-shrinking changes on the rebinding of the virus [25]. 

### 2.4. Bacterial and Fungal Cells

Direct bacteria imprinting and generation of recognition sites on polymeric matrices have demonstrated to be practicable [26]. It belongs to one type of cell-imprinted polymers. The recognition mechanism can give credit to the diversity of bacteria cells in shape (e.g., round-shaped *Staphylococcus aureus* and the rod-shaped *Escherichia coli* (*E. coli*)), the uniform size of the same bacteria and the relatively rigid cell wall, which enable size and shape-dependent physical space matching. More importantly, chemical recognition based on the multiple interactions between the cell surface and MIPs/SIPs is essential to recognizing bacteria cells. Ren and Zare [27] developed the bacteria cell-imprinted polydimethylsiloxane (PDMS) to investigate the role of chemical recognition (Figure 4B). The results showed that cell imprinted PDMS with methylsilane groups results in a cavity, thus losing much of its ability to capture the imprinted bacteria, although the shapes of the imprints were shown to be hardly affected which was proved by atomic force field microscopy. Hence, employing suitable functional groups or monomers to form efficient chemical interactions between MIPs/SIPs and the bacterial cell surface is a more important factor for cell-imprinting. Other studies also revealed that chemical recognition plays a dominant role in bacteria recognition. Phenylboronic acid (PBA) groups can significantly improve bacteria affinity of the MIPs with controllable bacteria recognition due to the reversibility between PBA and cis-diol groups of glycan chains presented on the bacterial surface [28]. Besides PBA groups, carbohydrate polymers like chitosan, which exhibits affinity for various bacteria strains, were also applied to create excellent MIPs matrices with high bacteria affinity [29].

The factors influencing yeast cells (*Saccharomyces cerevisiae*) recognition by SIPs were studied by means of spectroscopic and microscopy techniques. The results indicated that cell imprinting creates selective binding sites on the surface of the SIPs layer in the form of binding cavities that match the cells in shape and size. Furthermore, it demonstrated that the incorporated phospholipids significantly enhance cell adhesion to the SIPs. The role of phospholipids in the SIP recognition mechanism is mediated by long-range hydrophobic forces [7]. 

## 3. Preparation of MIPs/SIPs for Electrochemical Biosensor

Various methods have been applied for the production of MIPs/SIPs on electrodes to prepare electrochemical biosensors. Generally, they can be synthesized by three main steps: (i) assembly of functional monomer and template, (ii) polymerization of monomer-template complex with cross-linkers, porogen, and initiators under photo-/thermal/electrical conditions, and (iii) template removal to reveal binding microcavities that are highly specific to the template [31]. Standard free radical polymerization and sol-gel process are usually used. Free radical polymerization can be further categorized into bulk, multi-step swelling, suspension, emulsion, seed, and precipitation polymerizations based on their synthesis methods [32,33,34]. As a result, the microcavities that resemble the original template molecules in terms of size, shape, and orientation are generated in the polymer matrix, like the “lock-and-key”. Morphology of the polymer is determined by various factors, including polymer reaction time, the amount of pre-polymer, and porogenic solvent. 

A broad range of markers associated with infectious diseases such as antibiotics [35], lipopolysaccharides [36], nucleotides [37], toxin proteins [38,39], virus [40,41], bacteria [42,43], and fungi [7] cells have been successfully used as templates in synthesizing MIPs/SIPs. Gast et al. [12] highlighted synthesis strategies for virus imprinted polymers. Nowadays, double-templates [44,45] and multi-templates [46] methods have been developed, which makes MIPs/SIPs based-biosensors able to detect more target analytes in one complex sample.

The choice of a functional monomer is particularly essential to create highly specific microcavities for the templates. Interestingly, Su et al. [47] used computer-assisted molecular simulation calculations to select the suitable functional monomer and solvent for the template molecule. MAA is reported as the functional monomer which can form desirable pore shape and structure [48], meanwhile, it can be hydrogen bond based acceptor and donor [49]. Other monomers used in MIPs/SIPs synthesis include sulphonic acids (e.g., 2-acrylamido-2-methylpropane sulphonic acid), carboxylic acids (e.g., acrylic acid, vinylbenzoic acid), and heteroaromatic bases (e.g., vinylpyridine, vinylimidazole) were summarized by Choi and coworkers [33]. Typically, electropolymerizable monomers for the preparation of MIPs/SIPs were highlighted by Crapnell and coworkers [50]. MAA, polyvinylpyrrolidone (PVP), dimethylamino ethyl methacrylate (DMAEMA), and polyamine (PA) are usually used for bacteria imprinting to improve the recognition affinity for bacteria [30]. 

The crosslinker is another important component of MIPs/SIPs. It is responsible for the morphology and stability of imprinted binding sites. Ethylene glycoldimethacrylate (EGDMA), divinylbenzene (DVB), and trimethylolpropane trimethacrylate (TRIM) are the most reported cross-linkers [33]. The most common crosslinker for bacteria imprinting are polydimethylsiloxane (PDMS), polyacrylate, silica (SiO_2_), and polyurethane (PU) [30].

Most recently, the combination of nanoparticles with MIPs/SIPs to enhance the performance of electrochemical biosensors is a popular topic. Noble metal nanoparticles (such as Au, Ag, Pt, Pd, etc.), metal oxide nanomaterials (such as TiO_2_, Fe_2_O_3_, etc.), and carbon nanomaterials (such as carbon nanotubes, graphene, etc.) distinctly offer many unique advantages [51]. 

### 3.1. Deposition or Spin Coating on Electrodes

Deposition and spin coating are two simple methods for preparing MIPs/SIPs modified electrode. Tancharoen et al. [52] used spin coating method to prepare a SIPs for Zika virus (ZIKV) detection. In their procedures, a certain amount of the prepolymer−graphene oxide mixture was coated on a 1 × 1 cm^2^ gold electrode before spinning at 1000 rpm for 10 s to remove excess prepolymer. Subsequently, the ZIKV template was dispersed on the composite film and exposed to UV light before keeping in an oven at 65 °C for 15 h to allow polymerization to occur. The proposed SIPs were obtained after removing the template from the composite polymer by washing in acetic acid and deionized water. 

### 3.2. Assembly by Self-Assembled Monolayers 

Self-assembled monolayers (SAMs) can be used to immobilize MIPs nanoparticles onto the gold surface. Unlike the in-situ synthesis of MIPs/SIPs on an electrode surface, the method dependent on SAMs includes two steps. Firstly, MIPs nanoparticles need to be prepared, then the MIPs nanoparticles can be fixed on a SAMs modified electrode by the covalent bond. The solid-phase synthesis method was used by Tothill’s research group to fabricate the MIPs nanoparticles, then the amine coupling chemistry was used to fix nano MIPs receptors strongly to the gold chip. The principle of this method depends on the activation of carboxyl groups on the gold surface by an EDC/NHS mixture which forms reactive succinimide esters [53,54].

### 3.3. Electropolymerization or UV Light-Induced Polymerization

**Figure 5:**
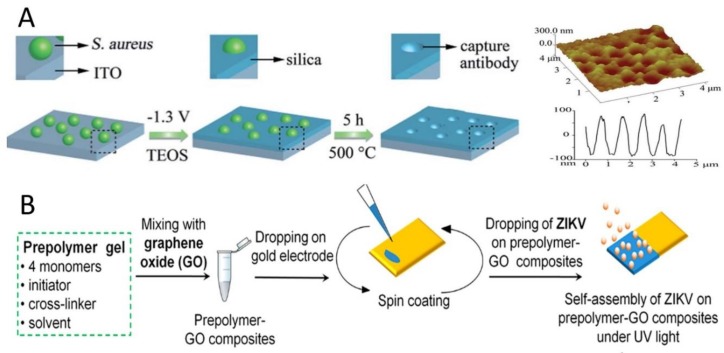
(**A**) Schematic preparation procedures for the artificial capture antibodies (cAbs), AFM images, and the corresponding height profiles of the cAbs. Reproduced from [55]—Published by The Royal Society of Chemistry. (**B**) Schematic preparation procedures for graphene oxide doped SIPs under UV light. Reproduced from [52]—Published by the American Chemical Society.

Electropolymerization is a simple and convenient deposition technique with a conductive polymer layer produced on an electrode surface combined with the template. The layer thickness can be controlled easily. The high-affinity binding sites can be formed by direct doping of templates into the polymer matrix [50]. Usually, the thickness of the film controlled by the electropolymerization conditions and can be characterized by electrical impedance spectroscopy (EIS) and cyclic voltammetry (CV). The charge-transfer resistance of the surface would be increased with the thicknesses added. It is mainly because the polymer holds a low-conductive nature. In order to fabricate a layer of effective MIPs/SIPs, it is critical to control the polymeric film so that it does not cover the whole template so that it can be removed easily and rebound later. If the MIPs/SIPs are too thin, there are no stable microcavities formed on the electrode. It also lowers sensitivity/affinity for the template, since a lower number of binding sites are available. In turn, if the MIPs/SIPs film is too thick, it may entrap the template within the polymeric matrix, hence make its removal/rebinding more difficult. Imprinted artificial capture antibodies (cAbs) for *Staphylococcus aureus (S. aureus)* were fabricated by electropolymerization [55]. By formation of a Schiff base linkage, *S. aureus* was fixed on the aldehyde functionalized ITO electrode surface first. Then, an in-situ electrochemically assisted polycondensation strategy was applied to deposit a silica film on the electrode surface around the *S. aureus*. Finally, a calcination treatment was used to achieve the cAbs. The cAbs with lots of regular cavities were observed after the removal of the template. The circular shape of the cavities was proven by images of higher magnification. The AFM image (Figure 5A) revealed that the depths of the cavities were ~160 nm. Apparently, the pathogen template was imprinted on the ITO surface successfully and the three-dimensional spheroidal architecture was observed. All the preparation process was also characterized by using electrochemical methods in the study. 

Tokonami et al. [56] applied a MIPs film consisting of overoxidized polypyrrole (OPPy) to recognize bacilliform bacteria specifically and rapidly. Polypyrrole (PPy) was synthesized using electrochemical polymerization combined with dielectrophoresis (DEP) technique. The DEP resulted in the *P. aeruginosa* being oriented in one direction, perpendicular to the film surface. The number of bacteria doped in the film was counted to be 1.8 × 10^9^ cm^−2^.

UV light-induced polymerization also can be used to prepare MIPs/SIPs on the electrode. It has been used to prepare SIPs-graphene oxide composites on the electrode for Zika virus (ZIKV) detection (Figure 5B) [52]. Idil et al. fabricated SIPs under UV-polymerization for *E. coli* detection [57]. 

### 3.4. Micro-Contact Imprinting 

**Figure 6:**
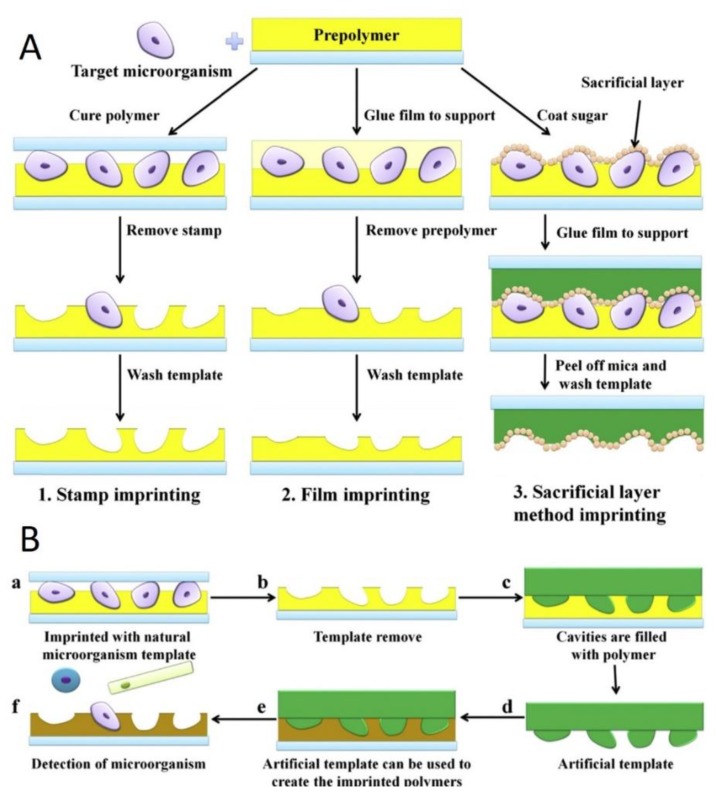
Schematic preparation procedures for three types of direct micro-contact imprinting (**A**) and indirect micro-contact imprinting (**B**). Reproduced from [26]—Published by Elsevier B.V.

The micro-contact imprinting approach is a soft lithography method that involves the conformal stamping of a template-immobilized layer in a specific pattern on a polymer surface (e.g., PU, PDMS, or SiO_2_), so that it is able to form shape-complementary recognition sites for relatively large templates on the surface. There are three main types of direct micro-contact imprinting methods: stamp imprinting, film imprinting, and sacrificial layer method imprinting (Figure 6A) [26]. It can also use an artificial template to generate the capturing of SIPs. This method is categorized as indirect micro-contact imprinting methods [58]. The preparation procedures are shown in Figure 6B. Stamp imprinting method was first used by Dickert et al. [59,60] to prepare SIPs to detect whole yeast cells. Recently, the micro-contact imprinting methods were considered as the most promising branch of MIPs/SIPs. 

## 4. Applications in Clinical Assays

### 4.1. Detection of Infectious Diseases Caused by Bacteria

Infectious diseases caused by bacteria are common in our life. MIPs/SIPs based electrochemical biosensors have been used as rapid diagnostic tools for these diseases. As a branch of MIPs/SIPs, CIPs are special for cell biomarkers. CIPs based electrochemical biosensor were reported for *Staphylococcus epidermidis* (*S. epidermidis*) detection [28]. 3-aminophenylboronic acid was used as a functional monomer for the electrochemical fabrication of the CIPs. EIS signal was shown to respond linearly to concentrations of *S. epidermidis* in the range of 10^3^–10^7^ cfu mL^−1^. MIPs fabricated by polyphenol was used as an artificial receptor for the detection of flagellar filaments from *Proteus mirabilis* by Khan and coworkers [61]. EIS and square wave voltammetry (SWV) were applied to measure the interaction of flagellar filaments with the MIPs that was fixed on their home-made paper-printed electrodes. Their results showed that the limit of detection (LOD) for the flagellar filaments was as low as 0.6 ng/mL. 

### 4.2. Detection of Infectious Diseases Caused by Viruses

MIPs/SIPs based biosensors have wide applications for the detection of virus in medical diagnostics. Malik and coworkers [62] summarized the state-of-the-art application of MIPs for virus detection. The detection performance for influenza, Dengue virus, Japanese encephalitis virus (JEV), human immunodeficiency virus (HIV), hepatitis A virus, hepatitis B virus, adenovirus, and picornaviruses were discussed. However, the studies cited in their review paper mainly used quartz crystal microbalance (QCM), surface plasmon resonance (SPR), fluorescence resonance energy transfer (FRET), and resonance light scattering (RLS) as transducers. In this section, the MIPs/SIPs based electrochemical biosensors for virus detection are emphasized. 

Human papillomavirus (HPV) is a group of more than 200 related viruses, some of which are spread through anal or vaginal sex. Long-lasting or chronic infections caused by HPV can induce cancer. Cai and coworkers [63] presented a MIPs based nano-sensor to detect human papillomavirus derived E7 protein. Analysis of EIS data revealed that the detection of E7 protein can be as low as sub pg L-1 levels. Notably, the human papillomavirus E6 protein (type-16) was not recognized by the E7 imprinted polymers. It shows outstanding specificity.

As a member of the Flaviviridae virus family, Zika virus usually infects human beings and typically causes a skin rash, conjunctivitis, red eyes, malaise, muscle and joint pain, headache, or mild fever. Recently, Tancharoen et al. [52] developed an electrochemical sensor based on SIPs and graphene oxide composite for Zika virus detection. The sensor was applied to detect virus in both PBS solutions and serum. In the PBS solution, LOD was found to be 2 × 10^−2^ PFU/mL in the presence of the dengue virus. For serum samples, dilution steps were added to reduce the background signal. The LOD found to be 2 × 10^−3^ in 10% serum samples and 5 × 10^−2^ PFU/mL (10~250 RNA copies/mL) in 1% serum samples. Generally, the lowest LOD in real samples should be 6000 (~10^4^) particles (or ~10^−3^ PFU) per mL. This performance is sufficient for Zika virus detection in practical applications.

Acquired immune deficiency syndrome (AIDS) is a severe infectious disease caused by HIV. HIV is a member of retroviruses, it is disseminated mainly by contaminated blood transfusions, unprotected sex, and others. Ma et al. [64] developed an electrochemical biosensor based on multi-walled carbon nanotubes modified MIPs for the detection of HIV-p24. They proved that MIPs have a specific recognition capacity for HIV-p24. The linear range was found to be from 1.0 × 10^−4^ ng cm^−3^ to 2.0 ng/cm^−3^. The LOD was tested to be 0.083 pg/cm^3^. The reported biosensor showed excellent selectivity and stability. It was successfully used for the detection of HIV-p24 in a human serum sample.

## 5. Conclusion and Look into the Future

Molecular imprinting is an attractive technology used to create selective recognition sites within a polymer network. MIPs/SIPs as tailor-made biomimetic materials have the obvious priority over other recognition elements. The major advantages are their robustness, long-term stability, and cost-effectiveness, which cannot be obtained by fragile biomolecules. In this review, applications of MIPs and SIPs based electrochemical biosensors are focused on, especially in the detection of infectious diseases. Recognition mechanisms, preparation methods, and application performance of MIPs/SIPs were discussed. Although tremendous progress has been achieved, there still exist several challenges. The most important one is that the sensitivity (Table 1) and selectivity need further improvement since MIPs/SIPs do not always possess properties comparable to antibodies. In this case, more functional monomers are worth exploring to promote chemical recognition. Another strategy is using nanopatterned electrodes as the transducer. The design and application of nanopatterned electrodes could promote MIPs/SIPs to generate more effective cavities with excellent spatial matching effect. Moreover, in the era of artificial intelligence, using machine learning to design MIPs/SIPs and improve the recognizing ability of MIPs/SIPs based electrochemical biosensors is very promising.

Until now, few studies explored the recognition mechanism of MIPs/SIPs and larger bioparticles (viruses and bacteria). Research on the exact mechanisms behind target recognition should be emphasized because that can lead to an in-depth understanding, which will eventually help in designing MIPs/SIPs and electrochemical biosensors with even higher selectivity, sensitivity, and accuracy.

## Figures and Tables

**Table 1:** Analytical performance of MIPs/SIPs based electrochemical biosensors for infectious diseases.

Analytes	Preparation Methods of MIPs/SIPs	Device/Indicator	Label/Label Free	Method	LOD	LR	Ref.
N-acyl-homoserine-lactones (AHLs)	MMIPs: Fe3O4@ SiO2-MIP	MGCE/ [Fe(CN)_6_]^3−/4−^	Label free	DPV	10^−10^ M	2.5 × 10^−9^–10^−7^ M	[21]
Bacterial surface proteins	3-aminophenol electropolymerization	SPEs-SWCNTs/ [Fe(CN)_6_]^3−/4−^	Label free	EIS	0.60 nM	NR	[65]
Bacterial flagellar filaments	Phenol electropolymerization	PPE/[Fe(CN)_6_]^3−/4−^	Label free	SWV	0.6 ng mL^−1^	0.01–100 μg mL^−1^	[61]
Staphylococcus epidermidis	3-APBA electropolymerization	GE//[Fe(CN)_6_]^3−/4−^	Label free	EIS	NR	10^3^–10^7^ CFU mL^−1^	[28]
*E. coli* O157:H7	PDA-SIPs	N-GQDs	Label	ECL	8 CFU mL^−1^	10–10^7^ CFU mL^−1^	[66]
*E. coli*	UV-polymerization	NR	Label free	Capacitance	70 CFU mL^−1^	1.0 × 10^2^–1.0 × 10^7^ CFU mL^−1^	[57]
*Bacillus cereus* spores	Pyrrole electropolymerization	CPE/[Fe(CN)_6_]^3−/4−^	Label free	CV	10^2^ CFU mL^−1^	10^2^–10^5^ CFU mL^−1^	[67]
*Zika virus*	Prepolymer-GO composites under UV light	SPGE//[Fe(CN)_6_]^3−/4−^	Label free	CV/EIS	∼10^−^^3^ PFU	10^−3^–10^2^ PFU mL^−1^	[52]
HIV-1 gene	Directly electropolymerization of phenylenediamine	ITO electrode/EsNCs	Label	ECL	0.3 fM	3.0 fM–0.3 nM	[37]
HIV-p24	polymerization using AAM as functional monomer, MBA as crosslinking agent and APS as initiator.	GCE		DPV	0.083 pg mL^−1^	1.0 × 10^−4^–2 ng mL^−1^	[64]
Aflatoxin B1	PATP-AuNPs electropolymerization	GE/[Fe(CN)_6_]^3−/4−^	Label free	LSV	3 fM	3.2 fM–3.2 µM	[23]

3-APBA: 3-aminophenylboronic acid. AAM: acrylamide. APS: ammonium persulfate. CPE: carbon paste electrode. CV: cyclic voltammetry. ECL: electrochemiluminescence. EsNCs: Europium sulfide nanocrystals. GCE: glassy carbon electrode. GE: gold electrode. LOD: limit of detection. LR: linear range. LSV: linear sweep voltammetry. MBA: N,N′-methylenebisacrylamide. N-GQDs: nitrogen-doped graphene quantum dots (N-GQDs). NR: not reported. PDA: polydopamine. PPE: paper-printed electrodes. SIPs: surface imprinted polymers. SPGE: screen-printed gold electrode.
